# FLAMSA-RIC for Stem Cell Transplantation in Patients with Acute Myeloid Leukemia and Myelodysplastic Syndromes: A Systematic Review and Meta-Analysis

**DOI:** 10.3390/jcm8091437

**Published:** 2019-09-11

**Authors:** Weerapat Owattanapanich, Patompong Ungprasert, Verena Wais, Smith Kungwankiattichai, Donald Bunjes, Florian Kuchenbauer

**Affiliations:** 1 Division of Hematology, Department of Medicine, Faculty of Medicine Siriraj Hospital, Mahidol University, Bangkok 10700, Thailand; 2 Clinical Epidemiology Unit, Department of Research and Development, Faculty of Medicine Siriraj Hospital, Mahidol University, Bangkok 10700, Thailand; 3 Department of Internal Medicine III, University Hospital of Ulm, 89081 Ulm, Germany; 4 Vancouver General Hospital, L/BMT Program of British Columbia, Vancouver, BC V5Z 1M9, Canada; 5 Terry Fox Laboratory, British Columbia Research Centre, Vancouver, BC V5Z 1L3, Canada

**Keywords:** FLAMSA, reduced-intensity, acute myeloid leukemia, myelodysplastic syndrome

## Abstract

Reduced-intensity conditioning (RIC) regimens are established options for hematopoietic stem cell transplantation (HSCT) for patients with acute myeloid leukemia (AML) and myelodysplastic syndrome (MDS). However, the efficacy of RIC regimens for patients with high-risk disease is limited. The addition of a fludarabine, amsacrine, and cytarabine (FLAMSA)-sequential conditioning regimen was introduced for patients with high-risk MDS and AML to combine a high anti-leukemic activity with the advantages of RIC. The current systematic literature review and meta-analysis was conducted with the aim of identifying all cohort studies of patients with AML and/or MDS who received FLAMSA-RIC to determine its efficacy and toxicity. Out of 3044 retrieved articles, 12 published studies with 2395 overall patients (18.1–76.0 years; 96.8% AML and 3.2% MDS; follow-up duration of 0.7–145 months; 50.3% had active AML disease before HSCT) met the eligibility criteria and were included in the meta-analysis. In the pooled analysis, the 1- and 3-year overall survival (OS) rates were 59.6% (95% confidence interval (CI), 47.9–70.2%) and 40.2% (95% CI, 28.0–53.7%), respectively. The pooled 3-year OS rate of the patients who achieved CR1 or CR2 prior to HSCT was 60.1% (95% CI, 55.1–64.8%) and the percentage of those with relapse or refractory disease was 27.8% (95% CI, 23.3–32.8%). The pooled 3-year leukemia-free survival (LFS) rate was 39.3% (95% CI, 26.4–53.9%). Approximately 29% of the patients suffered from grades 2–4 acute graft-versus-host disease (GVHD), while 35.6% had chronic GVHD. The pooled 1- and 3-year non-relapse mortality (NRM) rates were 17.9% (95% CI, 16.1–19.8%) and 21.1% (95% CI, 18.8–23.7%), respectively. Our data indicates that the FLAMSA-RIC regimen is an effective and well-tolerated regimen for HSCT in patients with high-risk AML and MDS.

## 1. Introduction

Reduced-intensity conditioning (RIC) regimens were initially introduced to reduce the adverse effects associated with myeloablative conditioning (MAC) and to improve the chance of successful hematopoietic stem cell transplantation (HSCT), especially in elderly and frail patients [1]. However, the efficacy of RIC regimens for patients who do not achieve a complete remission (CR) is limited [2]. The combination of fludarabine, amsacrine, and cytarabine (FLAMSA)-polychemotherapy with RIC was initially adopted by Schmid et al. for patients with high-risk myelodysplastic syndrome (MDS) and acute myeloid leukemia (AML) [3,4,5] to combine high anti-leukemic activity with the advantages of RIC. Although several variations have been published [6,7,8,9,10,11,12,13,14,15,16], the ‘classic’ FLAMSA-RIC regimen consists of fludarabine, amsacrine, and cytarabine, followed by RIC with 4-Gy total body irradiation (TBI), high-dose cyclophosphamide (Cy), antithymocyte globulin (ATG), and prophylactic donor lymphocyte infusions (DLI) if indicated. Because of initially promising data, especially in poor prognosis AML patients, FLAMSA-RIC was adopted by many transplantation centers, and variations that included busulfan (Bu) or treosulfan were established. The current systematic review and meta-analysis was conducted with the aim of identifying all cohort studies that have investigated the efficacy and toxicity of the FLAMSA-RIC regimen and summarize their results.

## 2. Materials and Methods

### 2.1. Data Sources and Searches

Three investigators (W.O., P.U. and S.K.) independently searched for published articles indexed in MEDLINE and EMBASE databases from their inception to May 2019. The search strategy is available as Supplementary Data 1. The references of the included studies were also manually reviewed for additional eligible studies. This study was undertaken in accordance with the Preferred Reporting Items for Systematic Reviews and Meta-Analyses (PRISMA) statement, which is available as Supplementary Data 2 [17].

### 2.2. Selection Criteria and Data Extraction

Studies included in this meta-analysis were cohort studies (either prospective or retrospective) of patients with AML and/or MDS who received FLAMSA-RIC regimens, which reported our primary outcomes of interest (overall survival (OS) and/or leukemic-free survival (LFS) rates). The secondary outcomes of interest, which included non-relapse mortality (NRM) rate, relapse rate (RR), full chimerism rate, grades 2–4 acute GVHD (aGVHD) rate, and chronic GVHD (cGVHD) rate were also collected for analysis but were not part of the inclusion criteria. Assessment of the eligibility of each study was independently conducted by three investigators. In the event of opposing decisions regarding a study’s eligibility, the study in question was reviewed by the three investigators together and the final determination was reached by mutual consensus.

### 2.3. Definition of Treatment Response and Outcome

Complete remission (CR) was defined as bone marrow blasts of < 5%, the absence of circulating blasts and blasts with Auer rods, the absence of an extramedullary disease, an absolute neutrophil count (ANC) of ≥ 1.0 × 10^9^/L and a platelet count of ≥ 100 × 10^9^/L [8]. Refractory AML was defined as failure to achieve CR following induction or salvage chemotherapy. Relapse AML was defined as recurrence of disease after CR. Overall survival (OS) rate was defined as the percentage of patients who were still alive at the time of interest (such as at 1 year after transplantation). Leukemia-free survival (LFS) rate was defined as the percentage of patients who were still alive and did not have leukemia at the time of interest. Non-relapse mortality (NRM) was defined as any death without previous relapse or progression.

### 2.4. Statistical Analysis

All data analyses were performed using the Comprehensive Meta-Analysis program, version 2.2 (Biostat, Englewood, NJ, USA). Using a standardized data extraction algorithm, two authors (W.O. and P.U.) extracted and tabulated all data from each study. The pooled rates and 95% confidence interval of OS rate, LFS rate, NRM rate, relapse rate, full chimerism rate, aGVHD rate, and cGVHD rate were calculated using the DerSimonian-Laird random-effect model with double arcsine transformation [18]. A random-effect model, rather than fixed-effect, was used because of the high likelihood of between-study heterogeneity. Cochran’s Q test and I^2^ statistic were used to determine the between-study heterogeneity. I^2^ statistic quantified the proportion of total variation across studies that is due to heterogeneity rather than chance. An I^2^ value of 0% to 25% represents insignificant heterogeneity, greater than 25% but less than or equal to 50% represents low heterogeneity, greater than 50% but less than or equal to 75% represents moderate heterogeneity, and greater than 75% represents high heterogeneity [19].

## 3. Results

**Figure 1:**
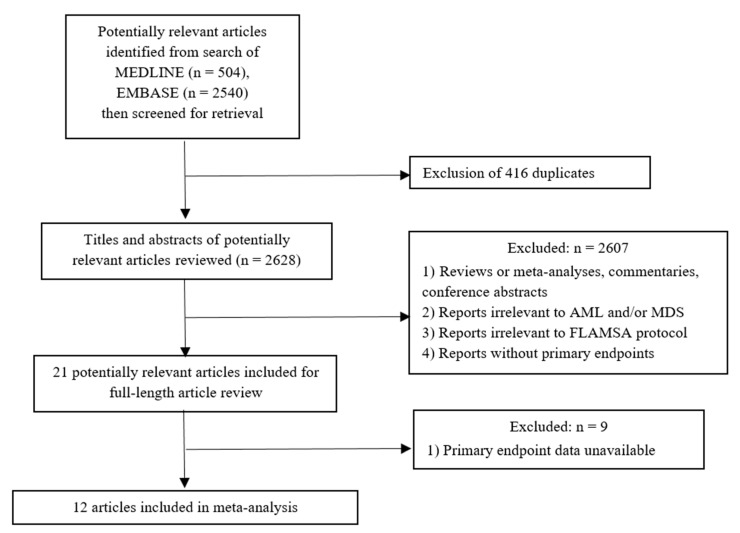
Flowchart of literature review process.

The search strategy yielded 3044 potentially relevant articles (504 articles from MEDLINE and 2540 from EMBASE). After exclusion of 416 duplicated articles using the EndNote X8 software, 2628 articles underwent title and abstract review. A total of 2607 articles were excluded at this stage as they did not meet the inclusion criteria based on type of article, study design, subjects and interventions used. A total of eighteen articles underwent full-text review and 9 of them were excluded because they did not report the primary outcomes of interest. Finally, 12 studies fulfilled the eligibility criteria and were included in the meta-analysis [3,6,7,8,9,10,11,12,13,14,15,16]. A manual review of the bibliography of the included studies, and some selected review articles, did not yield any additional eligible studies. Figure 1 summarizes the literature review and identification process. The main characteristics of the included studies are described in Table 1.

### 3.1. Baseline Patient Characteristics

A total of 12 studies with 2395 patients receiving FLAMSA-RIC regimen were included in this meta-analysis. 52.2% were male, age ranged from 18.1 to 76.0 years (46% were age 55 years or older). AML was by far the most common underlying hematological disease (70.6% de novo AML and 26.2% secondary AML). Only 3.2% of the analyzed patients had high-risk MDS. Thirty-seven per cent of the patients were in CR1, 11.4% of the patients were in CR2, and 50.3% of the patients had active AML prior to HSCT (49.9% relapse and/or refractory disease and 0.4% untreated AML). Baseline clinical characteristics of those patients are summarized in Table 2.

### 3.2. FLAMSA Variations, Stem Sources, and GVHD Prophylaxis

The FLAMSA regimen consists of fludarabine (30 mg/m^2^; total dose 120 mg/m^2^), amsacrine (100 mg/m^2^; total dose 400 mg/m^2^), and cytarabine (2 g/m^2^; total dose 8 g/m^2^) therapy from days minus 12 to minus 9, followed by a three-day interval without therapy and RIC. Several RIC protocols were included in this meta-analysis: (1) 4 Gy TBI plus Cy, (2) Bu/Cy, (3) treosulfan/Cy, (4) melphalan (Mel), (5) fludarabine (Flu)/Bu, (6) Bu alone, or (7) Mel/thiotepa. Almost all patients received rabbit anti-thymocyte globulin (rATG; 10–20 mg/kg from day minus 4 to day minus 2, according to donor type). Details of all included FLAMSA-RIC regimens, GvHD prophylaxis, and prophylactic donor lymphocyte transfusions are summarized in Supplementary Table S1.

Donors were investigated for human leukocyte antigen (HLA)-A, HLA-B, HLA-C, HLA-DRB1, and HLA-DQB1. In this study, 10/10 HLA-matched related (MRD), unrelated donors (MUD), 1–2 antigen/allele mismatched related (MMRD) and unrelated donors (MMUD) were included. The most frequent donors were MUD (52.5%), followed by MRD (33.3%), MMUD (13.7%) and MMRD (0.5%). The most frequent stem cell source (95.7%) were stem cells collected from peripheral blood. CD 34^+^ cell infusions ranged from 1.2 to 23.1 × 10^6^ cells/kg.

GVHD prophylaxis was available for 720 patients, most of them received cyclosporine A (CyA) plus mycophenolate mofetil (MMF; n = 573), followed by tacrolimus plus MMF (n = 112), CyA alone (n = 28), and CyA plus methotrexate (n = 7). Prophylactic donor lymphocyte transfusions were given if patients did not show any evidence for GVHD either at day 120 or 30 days after discontinuation of the immunosuppression [3,7,10,11,12].

### 3.3. Survival Outcome

**Figure 2:**
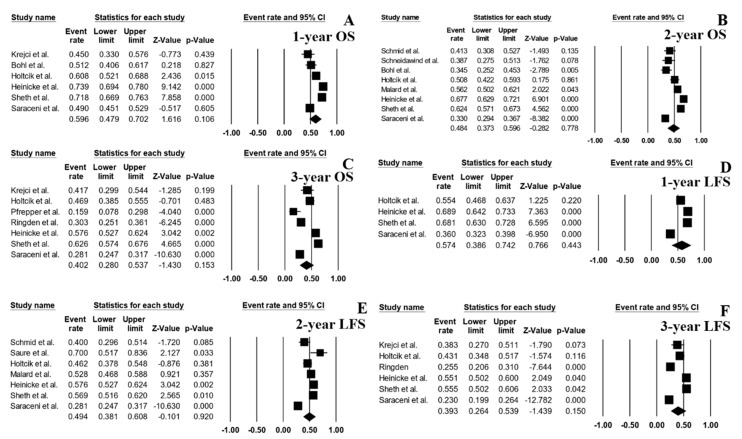
Forest plots of pooled estimates (95% confidence interval (CI)) for overall survival (OS) and leukemia-free survival (LFS) after hematopoietic stem cell transplantation (HSCT); (**A**): 1-year OS; (**B**): 2-year OS; (**C**): 3-year OS; (**D**): 1-year LFS; (**E**): 2-year LFS; (**F**): 3-year LFS.

**Figure 3:**
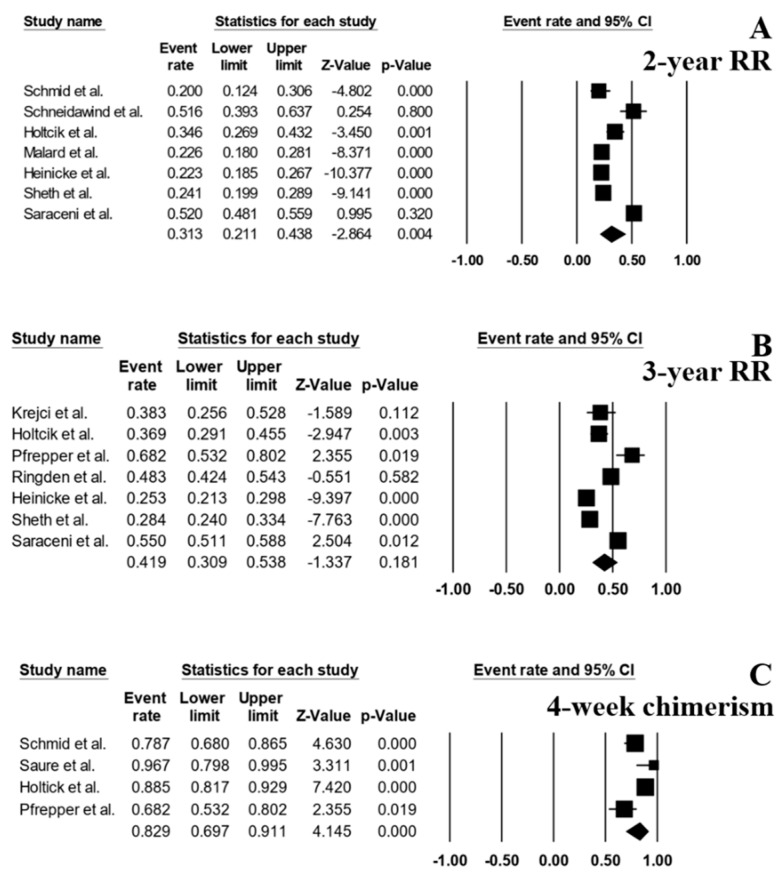
Forest plots of pooled estimates (95% CI) for relapse rate (RR) and outcome after HSCT; (**A**): 2-year RR; (**B**): 3-year RR; (**C**): full chimerism at 4 weeks after HSCT.

The follow-up period ranged from 0.7 to 145 months. The pooled 1-, 2- and 3-year OS rates were 59.6% (95% CI, 47.9–70.2%; I^2^ 94%; Figure 2A) [6,7,8,14,15,16], 48.4% (95% CI, 37.3–59.6%; I^2^ 96%; Figure 2B [3,6,8,10,13,14,15,16], and 40.2% (95% CI, 28.0–53.7%; I^2^ 96%; Figure 2C) [6,7,11,12,14,15,16], respectively. The pooled 1-, 2- and 3-year LFS were 57.4% (95% CI, 38.6–74.2%; I^2^ 98%; Figure 2D) [6,14,15,16], 49.4% (95% CI, 38.1–60.8%; I^2^ 95%; Figure 2E [3,6,9,13,14,15,16], and 39.3% (95% CI, 26.4–53.9%; I^2^ 97%; Figure 2F) [6,7,12,14,15,16], respectively. The pooled 2- and 3-year RR were 31.3% (95% CI, 21.1–43.8%; I^2^ 96%; Figure 3A) [3,6,10,13,14,15,16] and 41.9% (95% CI, 30.9–53.8%; I^2^ 95%; Figure 3B) [6,7,11,12,14,15,16], respectively.

A total of 4 studies [3,6,9,11] reported the rate of full chimerism at day +28 (defined as the presence of > 98% of HLA belonging to the donor) and the pooled rate across the 4 studies was 82.9% (95% CI, 69.7–91.1%; I^2^ 77%) (Figure 3C).

### 3.4. Complications of HSCT

**Figure 4:**
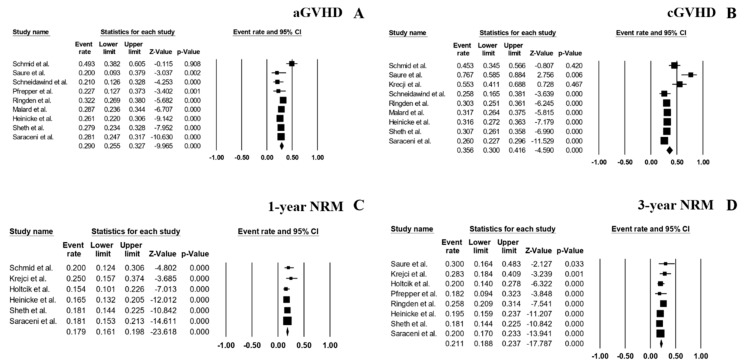
Forest plots of pooled estimates (95% CI) for complications after HSCT; (**A**): Acute graft-versus-host disease (aGVHD); (**B**): Chronic graft-versus-host disease (cGVHD); (**C**): 1-year non-relapse mortality (NRM); (**D**): 3-year NRM.

A total of 9 studies reported the rate of Grade 2–4 aGVHD. The pooled rate across those studies was 29.0% (95% CI, 25.5–32.7%; I^2^ 63%; Figure 4A) [3,9,10,11,12,13,14,15,16], whereas the pooled rate of cGVHD was 35.6% (95% CI, 30.0–41.6%; I^2^ 84%; Figure 4B), which was derived from 9 studies [3,7,9,10,12,13,14,15,16]. There was a slight increase in the rate of NRM for each year of follow-up with the pooled 1-year NRM rate of 17.9% (95% CI, 16.1–19.8%; I^2^ 0%; Figure 4C) [3,6,7,14,15,16], and the pooled 3-year NRM rate of 21.1% (95% CI, 18.8–23.7%; I^2^ 30%; Figure 4D) [6,7,9,11,12,14,15,16].

### 3.5. Subgroup Analysis

**Figure 5:**
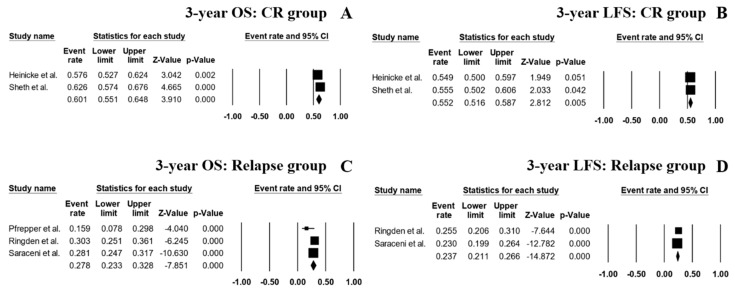
Forest plots of pooled estimates (95% CI) for outcomes of the patient’s subgroups; (**A**): 3-year OS of CR1 or CR2 patients; (**B**): 3-year LFS of CR1 or CR2 patients; (**C**): 3-year OS of acute myeloid leukemia (AML) patients with relapse and/or refractory disease; (**D**): 3-year LFS of high risk AML patients with relapse and/or refractory disease.

The pooled 3-year OS rate of the patients who achieved CR1 or CR2 prior to HSCT was 60.1% (95% CI, 55.1–64.8%; I^2^ 48%; Figure 5A) [14,15] and 3-year LFS was 55.2% (95% CI, 51.6–58.7%; I^2^ 0%; Figure 5B) [14,15]. As for the patients with relapse or refractory disease, the pooled 3-year OS and LFS rates were 27.8% (95% CI, 23.3–32.8%; I^2^ 47%; Figure 5C) [11,12,16] and 23.7% (95% CI, 21.1–26.6%; I^2^ 0%; Figure 5D) [12,16], respectively.

**Figure 6:**
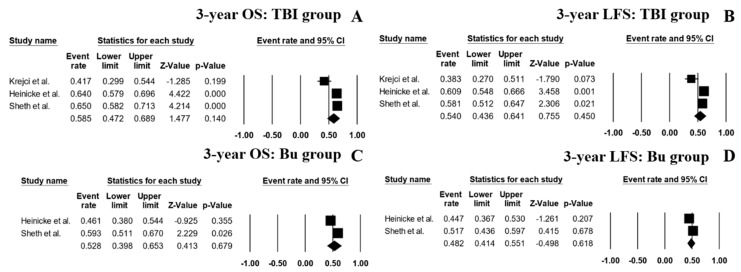
Forest plots of pooled estimates (95% CI) for outcome outcomes of the patient’s subgroups; (**A**): 3-year OS of patients receiving total body irradiation (TBI)-based conditioning regimen; (**B**): 3-year LFS of patients receiving TBI-based conditioning regimen; (**C**): 3-year OS of patients receiving busulfan-based conditioning regimen; (**D**): 3-year LFS of patients receiving busulfan-based conditioning regimen.

A subgroup analysis based on the reported conditioning regimens showed that the patients receiving a TBI-based regimen had a pooled 3-year OS rate of 58.5% (95% CI, 47.2–68.9%; I^2^ 82%; Figure 6A) [7,14,15] and a pooled 3-year LFS of 54.0% (95% CI, 43.6–64.1%; I^2^ 80%; Figure 6B) [7,14,15]. With regard to the patients receiving Bu-based regimens, the pooled 3-year OS rate was 52.8% (95% CI, 39.8–65.3%; I^2^ 80%; Figure 6C) [14,15] and the 3-year LFS was 48.2% (95% CI, 41.4–51.1%; I^2^ 29%; Figure 6D) [14,15].

### 3.6. Sensitivity Analysis

A sensitivity analysis was performed by excluding five studies by Ringden et al. [12], Malard et al. [13], Heinicke et al. [14], Sheth et al. [15] and Saraceni et al. [16] from the pooled analyses based on a concern over double-counting of patients. These five studies collected and reported data of patients who were treated at several medical centers and some centers also reported their own data separately (including the studies by Schmid et al. [3], Holtick et al. [6], Krejci et al. [7], Saure et al. [9], Schneidawind et al. [10], and Pfrepper et al. [11], which were included in this meta-analysis). The pooled 3-year OS, 3-year RR, and 3-year LFS were 34.9%, 47.2% and 41.6%, respectively. The new pooled results, after exclusion of the aforementioned studies, were fairly similar to the results of the original analysis as demonstrated in Supplementary Data 3.

## 4. Discussion

Although HSCT remains the most effective treatment option for AML, data from the National Cancer Data Base on patients with AML aged ≥ 61 years who were diagnosed between 2003 and 2012 found that only about 6% of older patients (n = 17,555) underwent HSCT [20]. RIC regimens were subsequently introduced with the aim of reducing adverse effects and making HSCT feasible for elderly and fragile patients. However, RIC regimens alone may not be sufficient for patients with high-risk features, such as persisting disease [21]. To overcome this limitation, FLAMSA-RIC was introduced in 2005 and has been adopted in many countries [3]. The current study is the first systematic review and meta-analysis to examine the efficacy and toxicity of the FLAMSA-RIC regimen for patients with AML and MDS. Recently published trials comparing MAC vs. RIC showed controversial results. For example, in the study of Scott et al., RIC led to significantly lower TRM but higher relapse rates compared with MAC, suggesting the use of MAC as the standard of care for fit patients with AML and MDS in CR [22]. In contrast, Bornhäuser et al. did not observe any difference between RIC and MAC with regards to relapse rate, OS and TRM for intermediate and high-risk AML patients [23]. We found a pooled 2-year OS rate of approximately 50%, which is lower than the reported results of Scott et al. (18-month OS: 76.4% MAC and 63.4% RIC) and Bornhäuser et al. (3-year OS: 61% vs. 58%), but similar or lower RR (FLAMSA: 2-year RR: 31.3%) compared to Scott et al. (18-month RR: 65.2%: MAC and 45.3%: RIC) and Bornhäuser et al. (3-year RR: 28% MAC and 26% RIC). In a retrospective analysis, Eapen et al. analyzed the impact of conditioning regimes with varying intensity on outcome in 2209 AML and MDS patients transplanted in complete remission [24]. The authors reported a higher 3-year RR with 46% with Flu/Bu2 and 56% with Flu/Bu2+ATG. In line with this finding, a recent retrospective EBMT analysis demonstrated a lower RR for FLAMSA-RIC compared to Flu/Bu2 in AML patients in CR1 and CR2 [14], suggesting that this patient group could benefit from FLAMSA-RIC. The clinical outcome was similar using either a TBI or Bu-based FLAMSA regimen. Considering the side-effects of TBI, FLAMSA-Bu appears to be an effective alternative to FLAMSA-TBI.

With regard to patients with active disease in this meta-analysis, the 3-year OS and LFS rates were 27.8% and 23.7%, respectively. Given, that only 46% of AML patients with induction failure respond to chemotherapy and that 52% with refractory disease die within 90 days [25], FLAMSA-RIC is a reasonable treatment option for this otherwise difficult to treat patient cohort. Interestingly, Bohl et al. recently showed a correlation between tumor load and outcome for FLAMSA-RIC, underscoring the relevance of a high tumor burden as one of the strongest negative predictors for treatment outcome after HSCT especially in relapsed or refractory AML patients [8]. Although Goyal et al. showed that the exclusive presence of extramedullary disease did not impact on outcome after HSCT [26], Bohl et al. noted that that FLAMSA-RIC followed by HSCT is not effective in patients with concurrent active bone marrow and extramedullary disease [8], which led to a change of practice in our institution.

The results may also suggest that the addition of FLAMSA to RIC may help to alleviate the aggressive behavior of the disease, which will ultimately help to improve survival outcome. Furthermore, the FLAMSA-RIC regimen did not appear to increase the risk of serious toxicity with the comparable 1-year NRM rate to those who received RIC regimens alone (17.9% versus 28% [27], respectively), as well as the comparable rate of grade 2–4 aGVHD events (29% versus 35% [27], respectively).

The limitations of this study are mainly due to the observational nature of the included primary studies. Varying event rates such as OS, LFS and RR among the included studies could attributed to differences in the analyzed patient cohorts and treatment protocols in each study. Furthermore, without a direct prospective head-to-head comparison, a conclusion on whether the FLAMSA-RIC regimen offers a superior survival benefit compared with RIC regimens alone among patients who do not achieve CR cannot be made. Randomized controlled trials comparing the FLAMSA-RIC regimen with RIC regimens alone are still needed. Ongoing randomized trials such as NCT01423175 and NCT00606723 comparing FLAMSA-RIC with alternative conditioning regimes will eventually prove its efficacy and toxicity as a standard conditioning protocol for high risk AML.

## 5. Conclusions

A FLAMSA-RIC regimen is an effective and well-tolerated regimen for HSCT in patients with AML and MDS, even among those who do not achieve complete remission.

## Figures and Tables

**Table 1:** Baseline patient characteristics of each included article.

References	No.	Sex (M/F)	Median Age (Years, Range)	Diseases	Disease Status	Stem Cell Source	Donor Source	CD34+ (×10^6^ cells/kg)	Study Period	Median Follow Up (Months, Range)	Type
Schmid et al. (2005) [4]	75	42/33	52.3 (18.5–65.8)	50 dAML, 15 sAML, 10 MDS	8 CR1, 8 CR2, 49 R/R, 10 MDS	61 PBSC, 14 BM	31 MRD, 30 MUD 6 MMRD, 8 MMUD	9.6	1999–2002	31.5 (13.6–47.6)	P
Saure et al. (2012) [9]	30	20/10	49 (36–66)	10 sAML, 20 MDS	10 untreated AML, 20 MDS	30 PBSC	13 MRD, 13 MUD 4 MMUD	7.7	2003–2010	28 (7–81)	P
Krejci et al. (2013) [7]	60	28/32	52 (20–63)	50 dAML, 10 sAML	34 CR1, 26 R/R	56 PBSC, 4 BM	15 MRD, 29 MUD, 16 MMUD	6.3	2006–2011	37 (10–69)	P
Schneidawind et al. (2013) [10]	62	34/28	55 (20–72)	35 dAML, 27 sAML	62 R/R	62 PBSC	11 MRD, 22 MUD 4 MMRD, 25 MMUD	5.4	2005–2012	17.5 (2.2–77.6)	R
Bohl et al. (2016) [8]	84	46/38	48.7	67 dAML, 17 sAML	13 CR1, 12 CR2, 59 R/R	NR	NR	NR	2000–2012	NR	R
Holtick et al. (2016) [6]	130	59/71	50.9 (19–73)	NR	47 CR1, 26 CR2, 57 R/R	127 PBSC, 3 BM	42 MRD, 64 MUD 1 MMRD, 23MMUD	7.07	2004–2015	37 (10–125)	R
Pfrepper et al. (2016) [11]	44	25/19	52 (21–65)	NR	44 R/R	44 PBSC	3 MRD, 27 MUD, 14 MMUD	NR	2006–2013	34 (6–71)	R
Ringden et al. (2016) [12]	267	131/136	51.7 (19.4–72.5)	NR	267 R/R	256 PBSC, 11 BM	77 MRD, 190 MUD	NR	NR	68.2 (2–157)	R
Malard et al. (2017) [13]	265	143/122	55 (19–76)	156 dAML, 109 sAML	216 CR1, 49 CR2	251 PBSC, 14 BM	74 MRD, 191 MUD	NR	2002–2014	46 (1–145)	R
Heinicke et al. (2018) [14]	399	206/193	(18–74.4)	NR	305 CR1, 94 CR2	379 PBSC, 20 BM	139 MRD, 198 MUD, 62 MMUD	NR	2005–2016	(0.7–121.5)	R
Sheth et al. (2019) [15]	348	179/169	(40.1–65)	294 dAML, 54 sAML	264 CR1, 84 CR2	330 PBSC, 18 BM	113 MRD, 182 MUD, 53 MMUD	NR	2007–2016	NR	R
Saraceni et al. (2019) [16]	631	336/295	51.5 (18.1–76)	NR	631 RR	616 PBSC, 15 BM	252 MRD, 268 MUD, 111 MMUD	NR	2005–2016	53 (4–35)	R

**Abbreviations**; BM bone marrow; CR1 complete remission after first induction therapy; CR2 complete remission after relapse; dAML denovo acute myeloid leukemia; F Female; M Male; MDS myelodysplastic syndromes; MRD match related donor; MMRD mismatch related donor; MMUD mismatch unrelated donor; MUD match unrelated donor; NR not reported; P prospectively; PBSC peripheral blood stem cell; R retrospectively; R/R relapse and/or refractory diseases; sAML secondary acute myeloid leukemia.

**Table 2:** Clinical characteristics of the patients from the included studies.

		Number of Patients (N = 2395)	Percent (%) or Range
Sex	Male	1249	52.2
	Female	1146	47.8
Age range in years		-	18.1–6.0
Diseases (n = 924)	dAML	652	70.6
	sAML	242	26.2
	MDS	30	3.2
Disease status (n = 2395)	CR1	887	37.0
	CR2	273	11.4
	R/R	1195	49.9
	Untreated AML	10	0.4
	High-risk MDS	30	1.3
Stem cell source (n = 2311)	PBSC	2212	95.7
	BM	99	4.3
Donor source (n = 2311)	MRD	770	33.3
	MUD	1214	52.5
	MMRD	11	0.5
	MMUD	316	13.7
CD 34+ in 10^6^ cells/kg (n = 357)		-	1.2–23.1
Follow up duration in months (n = 933)		-	0.7–145

**Abbreviations**; BM bone marrow; CR1 complete remission after first induction therapy; CR2 complete remission after relapse; dAML denovo acute myeloid leukemia; MDS myelodysplastic syndromes; MRD match related donor; MMRD mismatch related donor; MMUD mismatch unrelated donor; MUD match unrelated donor; PBSC peripheral blood stem cell; R/R relapse and/or refractory diseases; sAML secondary acute myeloid leukemia.

## Supplementary Materials

The following are available online at https://www.mdpi.com/2077-0383/8/9/1437/s1, Supplementary data 1: Search strategy, Supplementary data 2: The Preferred Reporting Items for Systematic Reviews and Meta-Analyses statement, Supplementary data 3: Sensitivity analysis of the odds of outcomes after HSCT; A: 3-year OS; B: 2-year relapse rate; C: 3-year relapse rate; D: 3-year LFS; E: aGVHD; F: cGVHD; G: 3-year NRM., Table S1: FLAMSA-RIC regimen, GvHD prophylaxis, and prophylactic donor lymphocyte transfusions in each study.

## Abbreviation

**Abbreviation**	**Full Name**
aGVHD	acute graft-versus-host disease;
AML	acute myeloid leukemia
Bu	busulfan
CI	confidence interval
cGVHD	chronic graft-versus-host disease
CR	complete remission
Cy	cyclophosphamide
CyA	cyclosporine A
Flu	fludarabine
GVHD	graft-versus-host disease
HLA	human leukocyte antigen
HSCT	hematopoietic stem cell transplantation
LFS	leukemia-free survival
MAC	myeloablative conditioning
MDS	MMUD mismatched unrelated donors
MRD	matched related donor
MUD	matched unrelated donors
NRM	non-relapse mortality
OS	overall survival
rATG	rabbit anti-thymocyte globulin
RIC	reduced-intensity conditioning
RR	relapse rate
TBI	total body irradiation

## References

[1] Slavin S., Nagler A., Naparstek E., Kapelushnik Y., Aker M., Cividalli G., Varadi G., Kirschbaum M., Ackerstein A., Samuel S. (1998). Non-MA stem cell transplantation and cell therapy as an alternative to conventional bone marrow transplantation with lethal cytoreduction for the treatment of malignant and nonmalignant hematologic diseases. Blood.

[2] Wais V., Kundgen L., Bohl S.R., von Harsdorf S., Schlenk R.F., Dohner K., Teleanu V., Bullinger L., Nguyen T.M., Drognitz K. (2018). Reduced-toxicity conditioning for allogeneic hematopoietic cell transplantation in elderly or comorbid patients with AML using fludarabine, BCNU and melphalan: Disease stage at transplant determines outcome. Bone Marrow Transplant..

[3] Schmid C., Schleuning M., Ledderose G., Tischer J., Kolb H.-J. (2005). Sequential Regimen of Chemotherapy, Reduced-Intensity Conditioning for Allogeneic Stem-Cell Transplantation, and Prophylactic Donor Lymphocyte Transfusion in High-Risk Acute Myeloid Leukemia and Myelodysplastic Syndrome. J. Clin. Oncol..

[4] Schmid C., Schleuning M., Schwerdtfeger R., Hertenstein B., Mischak-Weissinger E., Bunjes D., Harsdorf S.V., Scheid C., Holtick U., Greinix H. (2006). Long-term survival in refractory acute myeloid leukemia after sequential treatment with chemotherapy and reduced-intensity conditioning for allogeneic stem cell transplantation. Blood.

[5] Schmid C., Schleuning M., Hentrich M., Markl G.E., Gerbitz A., Tischer J., Ledderose G., Oruzio D., Hiddemann W., Kolb H.-J. (2008). High antileukemic efficacy of an intermediate intensity conditioning regimen for allogeneic stem cell transplantation in patients with high-risk acute myeloid leukemia in first complete remission. Bone Marrow Transplant..

[6] Holtick U., Shimabukuro-Vornhagen A., Chakupurakal G., Theurich S., Leitzke S., Burst A., Hallek M., von Bergwelt-Baildon M., Scheid C., Chemnitz J.M. (2016). FLAMSA reduced-intensity conditioning is equally effective in AML patients with primary induction failure as well as in first or second complete remission. Eur. J. Haematol..

[7] Krejci M., Doubek M., Dušek J., Brychtova Y., Racil Z., Navrátil M., Tomiska M., Horky O., Pospisilova S., Mayer J. (2013). Combination of fludarabine, amsacrine, and cytarabine followed by reduced-intensity conditioning and allogeneic hematopoietic stem cell transplantation in patients with high-risk acute myeloid leukemia. Ann. Hematol..

[8] Bohl S., Von Harsdorf S., Mulaw M., Hofmann S., Babiak A., Maier C.P., Schnell J., Hütter-Krönke L.-M., Scholl K., Wais V. (2016). Strong impact of extramedullary involvement in high-risk AML patients with active disease receiving the FLAMSA conditioning regimen for HSCT. Bone Marrow Transplant..

[9] Saure C., Schroeder T., Zohren F., Groten A., Bruns I., Czibere A., Galonska L., Kondakci M., Weigelt C., Fenk R. (2012). Upfront Allogeneic Blood Stem Cell Transplantation for Patients with High-Risk Myelodysplastic Syndrome or Secondary Acute Myeloid Leukemia Using a FLAMSA-Based High-Dose Sequential Conditioning Regimen. Biol. Blood Marrow Transplant..

[10] Schneidawind D., Federmann B., Faul C., Vogel W., Kanz L., Bethge W.A. (2013). Allogeneic hematopoietic cell transplantation with reduced-intensity conditioning following FLAMSA for primary refractory or relapsed acute myeloid leukemia. Ann. Hematol..

[11] Pfrepper C., Klink A., Behre G., Schenk T., Franke G.N., Jentzsch M., Schwind S., Al-Ali H.K., Hochhaus A., Niederwieser D. (2016). Risk factors for outcome in refractory acute myeloid leukemia patients treated with a combination of fludarabine, cytarabine, and amsacrine followed by a reduced-intensity conditioning and allogeneic stem cell transplantation. J. Cancer Res. Clin. Oncol..

[12] Ringdén O., Labopin M., Schmid C., Sadeghi B., Polge E., Tischer J., Ganser A., Michallet M., Kanz L., Schwerdtfeger R. (2017). Sequential chemotherapy followed by reduced-intensity conditioning and allogeneic haematopoietic stem cell transplantation in adult patients with relapse or refractory acute myeloid leukaemia: A survey from the Acute Leukaemia Working Party of EBMT. Br. J. Haematol..

[13] Malard F., Labopin M., Stuhler G., Bittenbring J., Ganser A., Tischer J., Michallet M., Kröger N., Schmid C., Huynh A. (2017). Sequential Intensified Conditioning Regimen Allogeneic Hematopoietic Stem Cell Transplantation in Adult Patients with Intermediate- or High-Risk Acute Myeloid Leukemia in Complete Remission: A Study from the Acute Leukemia Working Party of the European Group for Blood and Marrow Transplantation. Biol. Blood Marrow Transplant..

[14] Heinicke T., Labopin M., Schmid C., Polge E., Socié G., Blaise D., Mufti G.J., Huynh A., Brecht A., LeDoux M.-P. (2018). Reduced Relapse Incidence with FLAMSA–RIC Compared with Busulfan/Fludarabine for Acute Myelogenous Leukemia Patients in First or Second Complete Remission: A Study from the Acute Leukemia Working Party of the European Society for Blood and Marrow Transplantation. Biol. Blood Marrow Transplant..

[15] Sheth V., Labopin M., Canaani J., Volin L., Brecht A., Ganser A., Mayer J., Labussière-Wallet H., Bittenbring J., Shouval R. (2019). Comparison of FLAMSA-based reduced intensity conditioning with treosulfan/fludarabine conditioning for patients with acute myeloid leukemia: An ALWP/EBMT analysis. Bone Marrow Transplant..

[16] Saraceni F., Labopin M., Brecht A., Kröger N., Eder M., Tischer J., Labussière-Wallet H., Einsele H., Beelen D., Bunjes D. (2019). Fludarabine-treosulfan compared to thiotepa-busulfan-fludarabine or FLAMSA as conditioning regimen for patients with primary refractory or relapsed acute myeloid leukemia: A study from the Acute Leukemia Working Party of the European Society for Blood and Marrow Transplantation (EBMT). J. Hematol. Oncol..

[17] Moher D., Liberati A., Tetzlaff J., Altman D.G. (2009). Preferred Reporting Items for Systematic Reviews and Meta-Analyses: The PRISMA Statement. J. Clin. Epidemiol..

[18] Mathes T., Kuss O. (2018). A comparison of methods for meta-analysis of a small number of studies with binary outcomes. Res. Synth. Methods.

[19] Higgins J.P.T., Thompson S.G., Deeks J.J., Altman D.G. (2003). Measuring inconsistency in meta-analyses. BMJ.

[20] Bhatt V.R., Chen B., Gyawali B., Lee S.J. (2018). Socioeconomic and health system factors associated with lower utilization of hematopoietic cell transplantation in older patients with acute myeloid leukemia. Bone Marrow Transplant..

[21] De Lima M., Anagnostopoulos A., Munsell M., Shahjahan M., Ueno N., Ippoliti C., Andersson B.S., Gajewski J., Couriel D., Cortes J. (2004). Nonablative versus reduced-intensity conditioning regimens in the treatment of acute myeloid leukemia and high-risk myelodysplastic syndrome: Dose is relevant for long-term disease control after allogeneic hematopoietic stem cell transplantation. Blood.

[22] Scott B.L., Pasquini M.C., Logan B.R., Wu J., Devine S.M., Porter D.L., Maziarz R.T., Warlick E.D., Fernandez H.F., Alyea E.P. (2017). Myeloablative Versus Reduced-Intensity Hematopoietic Cell Transplantation for Acute Myeloid Leukemia and Myelodysplastic Syndromes. J. Clin. Oncol..

[23] Bornhäuser M., Kienast J., Trenschel R., Burchert A., Hegenbart U., Stadler M., Baurmann H., Schäfer-Eckart K., Holler E., Kröger N. (2012). Reduced-intensity conditioning versus standard conditioning before allogeneic haemopoietic cell transplantation in patients with acute myeloid leukaemia in first complete remission: A prospective, open-label randomised phase 3 trial. Lancet Oncol..

[24] Eapen M., Brazauskas R., Hemmer M., Perez W.S., Steinert P., Horowitz M.M., Deeg H.J. (2018). Hematopoietic cell transplant for acute myeloid leukemia and myelodysplastic syndrome: Conditioning regimen intensity. Blood Adv..

[25] Wattad M., Amlsg F.T.G.-A., Weber D., Döhner K., Krauter J., Gaidzik V.I., Paschka P., Heuser M., Thol F., Kindler T. (2017). Impact of salvage regimens on response and overall survival in acute myeloid leukemia with induction failure. Leukemia.

[26] Goyal S.D., Zhang M.J., Wang H.L., Akpek G., Copelan E.A., Freytes C., Gale R.P., Hamadani M., Inamoto Y., Kamble R.T. (2015). Allogeneic hematopoietic cell transplant for AML: No impact of pre-transplant extramedullary disease on outcome. Bone Marrow Transplant..

[27] Zhang Z.-H., Lian X.-Y., Yao D.-M., He P.-F., Ma J.-C., Xu Z.-J., Guo H., Zhang W., Lin J., Qian J. (2017). Reduced intensity conditioning of allogeneic hematopoietic stem cell transplantation for myelodysplastic syndrome and acute myeloid leukemia in patients older than 50 years of age: A systematic review and meta-analysis. J. Cancer Res. Clin. Oncol..

