# Supplementary material for: FLAMSA-RIC for Stem Cell Transplantation in Patients with Acute Myeloid Leukemia and Myelodysplastic Syndromes: A Systematic Review and Meta-Analysis
Source: J Clin Med. 2019 Sep 11;8(9):1437. doi: 10.3390/jcm8091437 (PMC6780116; doi:10.3390/jcm8091437)
Supplement: Supplementary file 1 [file jcm-08-01437-s001.zip › jcm-553005-supplematary/Supplementary Data 1.docx]

**Supplementary Data 1**

Search Strategy

**EMBASE**

1. 'acute myeloid leukemia'/exp OR 'acute myeloid leukemia'
2. 'myelodysplastic syndrome'/exp OR 'myelodysplastic syndrome'
3. #1 OR #2
4. 'fludarabine'/exp OR 'fludarabine'
5. 'amsacrine'/exp OR 'amsacrine'
6. 'cytarabine'/exp OR 'cytarabine'
7. #4 AND #5 AND #6
8. FLAMSA
9. 'reduced intensity conditioning'/exp OR 'reduced intensity conditioning'
10. #7 OR #8 OR #9
11. #3 AND #10

**MEDLINE**

1. myelodysplastic syndrome.mp. OR exp Myelodysplastic Syndromes/
2. acute myeloid leukemia.mp. OR exp Leukemia, Myeloid, Acute/
3. OR/#1-#2
4. fludarabine.mp.
5. cytarabine.mp. OR exp CYTARABINE/
6. amsacrine.mp. OR exp AMSACRINE/
7. FLAMSA
8. reduced intensity conditioning.mp.
9. #4 and #5 and #6
10. OR/#7-#9
11. #3 and #10
