# Supplementary material for: FLAMSA-RIC for Stem Cell Transplantation in Patients with Acute Myeloid Leukemia and Myelodysplastic Syndromes: A Systematic Review and Meta-Analysis
Source: J Clin Med. 2019 Sep 11;8(9):1437. doi: 10.3390/jcm8091437 (PMC6780116; doi:10.3390/jcm8091437)
Supplement: Supplementary file 1 [file jcm-08-01437-s001.zip › jcm-553005-supplematary/Supplementary Data 3.docx]

**
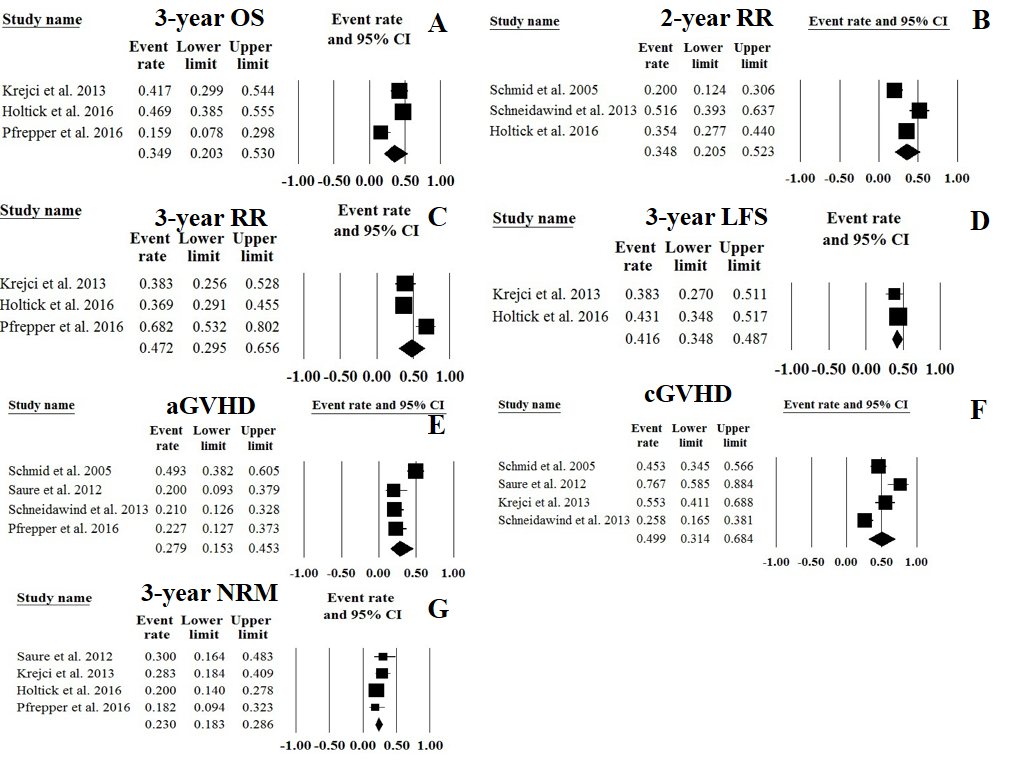
**

**Supplementary Data 3** Sensitivity analysis of the odds of outcomes after HSCT; **A**: 3-year OS; **B**: 2-year relapse rate; **C**: 3-year relapse rate; **D**: 3-year LFS; **E**: aGVHD; **F**: cGVHD; **G**: 3-year NRM
