# Supplementary material for: FLAMSA-RIC for Stem Cell Transplantation in Patients with Acute Myeloid Leukemia and Myelodysplastic Syndromes: A Systematic Review and Meta-Analysis
Source: J Clin Med. 2019 Sep 11;8(9):1437. doi: 10.3390/jcm8091437 (PMC6780116; doi:10.3390/jcm8091437)
Supplement: Supplementary file 1 [file jcm-08-01437-s001.zip › jcm-553005-supplematary/Supplementary Table S1.docx]

**Supplementary Table S1** FLAMSA-RIC regimen, GvHD prophylaxis, and prophylaxis donor lymphocyte transfusion in each study

| **References** | **FLAMSA-RIC protocol in each study** |
| --- | --- |
| Schmid et al. (2005)  [3] | ***FLAMSA regimen***: Flu 30 mg/m^2^, HD Ara-C 2 g/m^2^, and amsacrine 100 mg/m^2^ from days -12 to -9  ***RIC regimen***: After 3 days of rest, 4 Gy TBI on day -5, Cy (40 mg/kg with MRD, 60 mg/kg for MUD, MMRD, or MMUD) on days -4 and -3, and rATG (10 mg/kg for MRD, 20 m/kg for MUD, MMRD, or MMUD) from days -4 to day -2  ***GvHD prophylaxis****:*  CyA from day -1, and MMF (15 mg/kg bid), starting from day 0  In the absence of GvHD, MMF was discontinued by day +50 and CyA was tapered from days +60 to +90.  ***pDLTs***: The patients were in CR without evidence of GvHD at day +120 or 30 days after discontinuation of immunosuppression. The dose of pDLTs was 1 to 5x 10^6^ CD3^+^ cells/kg; in the absence of GvHD, pDLTs was repeated up to three times, using escalating cell doses (5-to10-fold increase/transfusion) at 4-to 6-week intervals. |
| Saure et al. (2012)  [9] | ***FLAMSA regimen***: Flu 30 mg/m^2^, HD Ara-C 2 g/m^2^, and amsacrine100mg/m^2^ from days -9 to -6  ***RIC regimen***:  After 2 to 3 days of rest, HD-Mel on day -2 (n=24), or Mel/Thio (10 mg/kg, n=6) on day -2 to -3  The dose of Mel was adapted to the patient’s age as follows: <50 years: 200 mg/m^2^, 50-60 years: 150 mg/m^2^, >60 years: 100 mg/m^2^.  rATG (10-20 mg/kg) from days -5 to day -3  ***GvHD prophylaxis****:*  tacrolimus from day -1 (aiming at concentrations 10-15 mg/ml), and MMF (10-15 mg/kg bid; starting 1 hour after HSCT from day 0)  In the absence of GvHD, MMF was halved on day +28 and discontinued on day +42 while tapering of tacrolimus was started between day+80 and day +100. The aim was to be off immunosuppressive therapy between day +120 and day +140.  ***pDLTs***: NR |
| Krejci et al. (2013)  [7] | ***FLAMSA regimen***: Flu 30 mg/m^2^, HD Ara-C 2 g/m^2^, and amsacrine 100 mg/m^2^ from days -12 to -9  ***RIC regimen***: After 3 days of rest, 4 Gy TBI on day -5, Cy (40 mg/kg with MRD, 60 mg/kg for MUD) on days -4 and -3, and fATG (10 mg/kg for MRD, 20 m/kg for MUD) from days -4 to day -2  ***GvHD prophylaxis****:*  CyA from day -1, and MMF (15 mg/kg bid), starting from day 0  CyA was tapered from days +60 to +90.  ***pDLTs***: The patients were in CR without evidence of GvHD at days ≥120 and at least 30 days after discontinuation of immunosuppression. The first dose of pDLTs was 5x 10^6^ CD3^+^ cells/kg; the second dose was 1×10^7^ CD3^+^ cells/kg; and the third dose was 5×10^7^ CD3^+^ cells/kg; using escalating cell doses at 4-to 6-week intervals. |
| Schneidawind et al. (2013)  [10] | ***FLAMSA regimen***: Flu 30 mg/m^2^, HD Ara-C 2 g/m^2^, and amsacrine 100 mg/m^2^ from days -12 to -9  ***RIC regimen***:  After 3 days of rest, Flu (30 mg/m2 on day -5 to -4)/Bu (0.8 mg/kg twice on day -6, and 0.8 mg/kg four times daily on day -5 to -4, n=12), or 4 Gy TBI on day -5 /Cy (60 mg/kg) on days -4 and -3 (n=31), or Bu (0.8 mg/kg once on day -6, 0.8 mg/kg four times daily on day -5, and 0.8 mg/kg three times on day -4) /Cy (60 mg/kg for MUD, MMRD, and MMUD or 40 mg/kg for MRD on days -3 to -2, n=19)  fATG (10 mg/kg for MRD, 20 m/kg for MUD, MMRD, or MMUD) from days -3 to day -1  ***GvHD prophylaxis****:*  CyA from day -1 (plasma level 200–250 ng/ml, n=8), or tacrolimus (10-15 ng/ml starting from day -1, n=54) combined with MMF (1 g twice daily, start day +1)with  In the absence of GvHD, MMF tapered 500 mg every week from day +30 and CyA was tapered from days +60 to +90.  ***pDLTs***: Patients received additional DLI in case of mixed chimerism, relapse, or disease progression. |
| Bohl et al. (2016)  [8] | ***FLAMSA regimen***: NR  ***RIC regimen***: NR  ***GvHD prophylaxis****:* NR  ***pDLTs***: NR |
| Holtick et al. (2016)  [6] | ***FLAMSA regimen***: Flu 30 mg/m^2^, HD Ara-C 2 g/m^2^, and amsacrine 100 mg/m^2^ from days -13 to -10  ***RIC regimen***: After 3 days of rest, treosulfan (10 g/m^2^/d) from day -6 to day -4, Cy (40 mg/kg/d for MRD, and 60 mg/kg/d for MUD) from day -3 to -2, and ATG (10 mg/kg/d with MRD, 20 mg/kg/d with MUD, MMRD, or MMUD) from day -3 to day -1  ***GvHD prophylaxis****:*  CyA from day -1 to +100 adjusted to serum level (250–350 ng/mL), and MMF (2 g/d), starting from day 0 to +30  In the absence of GvHD, MMF was tapered from day +30, and discontinued by day +50 whereas CyA was tapered from day +100, and discontinued from day +180.  ***pDLTs***: No prophylactic donor lymphocyte infusions were administered. |
| Pfrepper et al. (2016)  [11] | ***FLAMSA regimen***: Flu 30 mg/m^2^, HD Ara-C 2 g/m^2^, and amsacrine 100 mg/m^2^ from days -12 to -9, and ATG (2 mg/m^2^) from day −3 to day −1 when unrelated donors were used  ***RIC regimen***: After 3 days of rest,  4 Gy TBI on day -5, and Cy (120 mg/kg) on days -4 and -3 (n=10) or  Oral Bu (8 mg/m^2^) on day -5, and Cy (120 mg/kg) on days -4 and -3 (n=24)  ***GvHD prophylaxis****:*  CyA (5 mg/kg divided into 2 doses) from day -1, and MMF (15 mg/kg bid), starting from day 0  In the absence of GvHD, MMF was discontinued by day +50 and CyA was tapered from days +60 to +90.  ***pDLTs***: Prophylactic DLI was administered in four patients in remission, free of immunosuppression and without GvHD with increasing doses starting from day +120. |
| Ringden et al. (2016)  [12] | ***FLAMSA regimen***: Flu 30 mg/m^2^, HD Ara-C 2 g/m^2^, and amsacrine 100 mg/m^2^ from days -12 to -9, and ATG were given 239 cases.  ***RIC regimen***: After 3 days of rest,  4 Gy TBI /Cy 120 mg/kg (n = 213) or 2 Gy TBI (n = 1), or  Bu 6.4 mg/kg plus Cy 120 mg/kg (n = 40) or  Bu alone 6.4 mg/kg (n = 8) or  Mel 100 mg/m^2^ (n = 5)  ***GvHD prophylaxis****:*  CyA from day -1, and MMF (15 mg/kg bid), starting from day 0 (n=180) or  CyA alone (n=20) or  CyA plus a short course of methotrexate (n=7) or  Tacrolimus combined with MMF (n=28) or  Other regimens (n=31)  ***pDLTs***: DLTs were given to 35 patients as pre-emptive therapy, to 24 after relapse, and to two because of minimal residual disease in bone marrow. |
| Malard et al. (2017)  [13] | ***FLAMSA regimen***: Flu 30 mg/m^2^, HD Ara-C 2 g/m^2^, and amsacrine 100 mg/m^2^ from days -12 to -9, and ATG  ***RIC regimen***: After 3 days of rest,  4 Gy TBI /Cy 120 mg/kg (n=153) or  Bu 6.4 mg/kg plus Cy 120 mg/kg (n = 101)  ***GvHD prophylaxis****:*  CyA from day -1, and MMF (15 mg/kg bid), starting from day 0 (n=216) or  Other regimens (n=48)  ***pDLTs***: NR |
| Heinicke et al. (2018)  [14] | ***FLAMSA regimen***: Flu 30 mg/m^2^ x 4 days, HD Ara-C 2 g/m^2^ x 4 days, and amsacrine 100 mg/m^2^ x 4 days and ATG 3 x 10 mg/kg  ***RIC regimen***:  4 Gy TBI /Cy 2 x 40 mg/kg (n=258) or  Bu 8 x 0.8 mg/kg plus Cy 2 x 40 mg/kg (n = 141)  ***GvHD prophylaxis****:* NR  ***pDLTs***: DLTs were given to 58 patients as pre-emptive therapy and to 21 after relapse. |
| Sheth et al. (2019)  [15] | ***FLAMSA regimen***: Flu 30 mg/m^2^, HD Ara-C 2 g/m^2^, and amsacrine 100 mg/m^2^ from days -12 to -9  ***RIC regimen***:  4 Gy TBI day-3 to day-2 /Cy 120 mg/kg day-5 to day-4 (n=203) or  Bu 6 mg/kg day-3 to day-2 plus Cy 120 mg/kg day-5 to day-4 (n = 145)  ***GvHD prophylaxis****:* NR  ***pDLTs***: NR |
| Saraceni et al. (2019)  [16] | ***FLAMSA regimen***: NR  ***RIC regimen***: 4 Gy TBI /Cy or Bu/Cy  ***GvHD prophylaxis****:* NR  ***pDLTs***: NR |

**Abbreviations:** *(a)GVHD* (acute) graft-versus-host disease; *Ara-C* cytarabine; *ATG* antithymocyte globulin; *Bu* busulfan; *Cy* cyclophosphamide; *CyA* cyclosporine; *f* fresenius*; Flu* fludarabine; *HD* high-dose; *Mel* melphalan; *HSCT* hematopoietic stem cell transplantation; *MMF* mycophenolate mofetil*; MRD* match related donor; *MMRD* mismatch related donor; *MMUD* mismatch unrelated donor; *MUD* match unrelated donor; *NR* not reported; *pDLTs* prophylactic donor lymphocyte transfusions; *r* rabbit; *TBI* total-body irradiation; *Thio* thiotepa;
